# Regulation of phenylpropanoid biosynthesis by MdMYB88 and MdMYB124 contributes to pathogen and drought resistance in apple

**DOI:** 10.1038/s41438-020-0324-2

**Published:** 2020-07-01

**Authors:** Dali Geng, Xiaoxia Shen, Yinpeng Xie, Yusen Yang, Ruiling Bian, Yuqi Gao, Pengmin Li, Liying Sun, Hao Feng, Fengwang Ma, Qingmei Guan

**Affiliations:** 1 State Key Laboratory of Crop Stress Biology for Arid Areas/Shaanxi Key Laboratory of Apple, College of Horticulture, Northwest A&F University, Yangling, Shaanxi 712100 China; 2 State Key Laboratory of Crop Stress Biology for Arid Areas, College of Plant Protection, Northwest A&F University, Yangling, Shaanxi 712100 China

**Keywords:** Drought, Biotic

## Abstract

MdMYB88 and MdMYB124 have been demonstrated to be responsible for lignin accumulation in apple under drought stress. In this study, using a metabolomic approach, we identified differentially accumulated phenylpropanoid and flavonoid metabolites in *MdMYB88/124* transgenic RNAi plants under control and long-term drought stress conditions in apple roots. We confirmed the regulation of phenylalanine by MdMYB88 and MdMYB124 via UPLC-MS in apple roots under both control and drought conditions. Using Electrophoretic Mobility Shift Assay (EMSA) and ChIP-quantitative PCR (qPCR) analyses, we found that MdMYB88 positively regulates the MdCM2 gene, which is responsible for phenylalanine biosynthesis, through binding to its promoter region. Under long-term drought conditions, *MdMYB88/124* RNAi plants consistently accumulated increased amounts of H_2_O_2_ and MDA, while *MdMYB88* and *MdMYB124* overexpression plants accumulated decreased amounts of H_2_O_2_ and MDA. We also examined the accumulation of metabolites in the phenylpropanoid biosynthesis pathway in the leaves of *MdMYB88* and *MdMYB124* transgenic apple plants after long-term drought stress. We found that metabolites responsible for plant defense, including phenylpropanoids and flavonoids, accumulated less in the RNAi plants but more in the overexpression plants under both control and drought conditions. We further demonstrated that *MdMYB88/124* RNAi plants were more sensitive to *Alternaria alternata* f. sp. *mali* and *Valsa mali*, two pathogens that currently severely threaten apple production. In contrast, *MdMYB88* and *MdMYB124* overexpression plants were more tolerant to these pathogens. The cumulative results of this study provided evidence for secondary metabolite regulation by MdMYB88 and MdMYB124, further explained the molecular roles of MdMYB88 and MdMYB124 in drought resistance, and provided information concerning molecular aspects of their roles in disease resistance.

## Introduction

Previous research has shown that two atypical and paralogous MYB transcription factors, namely, MdMYB88 and MdMYB124, are responsible for freezing and drought stress tolerance of apple plants^1,2^. In addition, MdMYB88 and MdMYB124 can also regulate the biosynthesis of lignin under drought stress by directly targeting MdMYB46 and MdVND6, two important regulators of the biosynthesis of secondary cell wall components^2^. In plants, lignin biosynthesis occurs via the phenylpropanoid biosynthesis pathway^3^; therefore, it is compelling to know whether MdMYB88 and MdMYB124 regulate lignin biosynthesis via the regulation of phenylpropanoid biosynthesis. Additionally, whether the biosynthesis of other secondary metabolites is regulated by MdMYB88 and MdMYB124 is important to further understand their roles in stress responses.

The phenylpropanoid biosynthesis pathway begins with phenylalanine, which can be converted into aromatic compounds, including benzenoids, coumarins, flavonoids, hydroxycinnamates, and lignin^4^. These compounds often participate in plant development^5^ and plant-environment interactions^6^. Phenylalanine is synthesized from the shikimate pathway, which requires the participation of chorismate mutase (CM)^4,7^. In *Petunia hybrida* cv. Mitchell, two CMs at different loci are directly responsible for the biosynthesis of phenylalanine^7,8^. In addition, the expression level of *CM*s can substantially influence the accumulation of phenylalanine, which further affects the biosynthesis of phenylpropanoids in plants^9–13^.

Many phenylpropanoids and flavonoids are involved in the responses to oxidative stress^14–16^, drought stress^17^, and disease resistance^18–21^. In *Vitis vinifera*, genes related to phenylpropanoid metabolism have been found to be less induced and/or more repressed in drought-sensitive rootstocks^22^. Research has also shown that phenylpropanoids and flavonoids play critical roles in the response to ozone-induced oxidative stress, as relatively high contents of phenylpropanoids and flavonoids in *Fraxinus excelsior* L. lead to relatively low-ozone sensitivity^23^. In addition, phenylpropanoids play a prominent role in the response to drought-induced oxidative stress in *Platanus* × *acerifolia*. The great investment of *Platanus* × *acerifolia* in phenylpropanoid biosynthesis complements the decrease in insufficient amounts of antioxidants during drought stress^24^. Phenylpropanoids and flavonoids also participate in the response to *Alternaria alternata* f. sp. *mali*. Higher contents of phenylpropanoids and flavonoids can enhance the resistance of tomatoes to *A. alternata*^25^. The defense against *Valsa mali* in plants also requires phenylpropanoids and flavonoids^26^. It has been shown that transgenic *MdUGT88F1* (a key UDP-glucose:phloretin 2’-O-glucosyltransferase gene) apple plants with increased phloridzin have enhanced resistance to Valsa canker^27^.

Abiotic and biotic stresses, including drought and disease, have been threatening apple growth and production worldwide. Drought often results in reduced photosynthesis and solute transport, loss of turgidity, membrane lipid peroxidation, and other problems in apple^27^. Membrane lipid peroxidation during drought stress always leads to secondary oxidative stress^28^. An increase in the levels of reactive oxygen species (ROS), such as hydrogen peroxide (H_2_O_2_), is the most direct indicator of oxidative stress, and malondialdehyde (MDA) is the product of membrane lipid peroxidation^28^. Disease caused by *A. alternata* f. sp. *mali* and *Valsa mali* affect apple trees by infecting the leaves and young shoots and subsequently causing defoliation, which leads to premature fruit drop^29–32^. A number of studies have characterized genes important for the resistance of apple to drought and pathogens and have examined their potential use in the breeding of resistant apple^33–37^. MYB88 and MYB124 and their homologous genes in apple participate in the response to salt, cold, and drought stress^1,2,38–41^. In the current study, we revealed that MdMYB88 and MdMYB124 regulate the accumulation of metabolites involved in the phenylpropanoid pathway by directly regulating the expression of *MdCM2*, which contributes to pathogen defense and drought stress tolerance.

## Results

### MdMYB88 and MdMYB124 regulate secondary metabolite accumulation in apple roots

Previously, we determined that MdMYB88 and MdMYB124 regulate root lignin deposition under drought stress. Here, we carried out a secondary metabolic profiling analysis to identify additional secondary metabolites regulated by MdMYB88 and MdMYB124 under both control and drought conditions. We used an ultra-performance liquid chromatography-tandem mass spectrometry (UPLC-MS) method in conjunction with previously generated *MdMYB88/124* RNAi plants in which both *MdMYB88* and *MdMYB124* were knocked down^1^. The results showed that a total of 678 metabolites were detected with this approach. Among these metabolites, 58 were flavone metabolites, 158 were flavonoid metabolites (including 13 catechins and catechin derivatives), 15 were anthocyanins and anthocyanin derivatives, 33 were flavonols, 29 were flavonoid glycosides, 19 were flavanones, 19 were quinic and quinic derivatives, and 17 were coumarins and coumarin derivatives (Supplementary Table 1).

We first applied principal component analysis (PCA) and orthogonal partial least squares discriminant analysis (OPLS-DA) on these metabolites to discover the differences in metabolites within and between sample groups. The PCA and OPLS-DA results showed that the metabolites of different genotypes and treatments were significantly different and could be used for further analysis (Supplementary Fig. 1A, B). We then performed a normalized clustering analysis and Pearson’s correlation analysis to analyze the homogeneity of the biological replicates of each group (Supplementary Fig. 1C, D). The second biological replicate of GL-3 under the control conditions was removed due to a relatively low coefficient (<0.9).

**Fig. 1:**
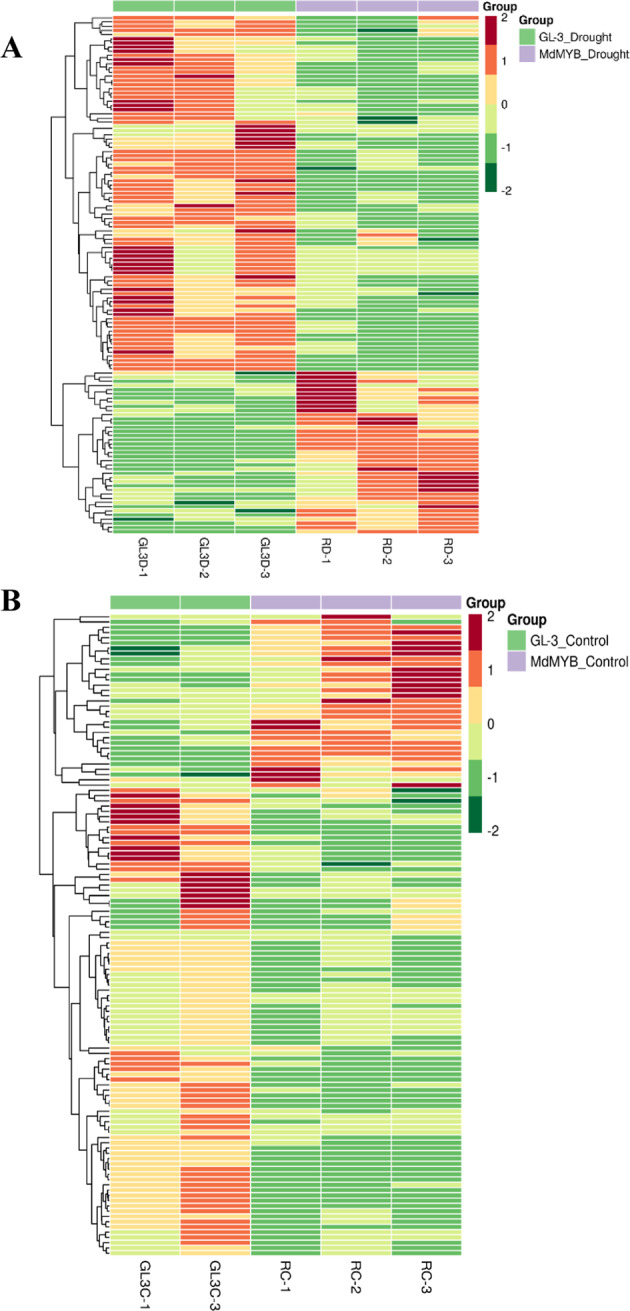
Heat map of differentially regulated metabolites. Heat map of differentially regulated metabolites in GL-3 and *MdMYB88/124* RNAi plants under long-term drought stress conditions (**a**) and control conditions (**b**)

To determine the differentially regulated metabolites, we performed a normalized clustering analysis (Fig. 1). Under the control conditions, 33 metabolites were found to be upregulated in the *MdMYB88/124* RNAi plants compared to those in the GL-3 plants. Furthermore, 89 metabolites were found to be downregulated in the *MdMYB88/124* RNAi plants compared to those in the GL-3 plants under the control conditions. Under drought conditions, 40 metabolites were found to be upregulated in the *MdMYB88/124* RNAi plants compared to GL-3 plants, and 99 metabolites were found to be downregulated. These results suggest that MdMYB88 and MdMYB124 regulate a broad range of secondary metabolites.

**Fig. 2:**
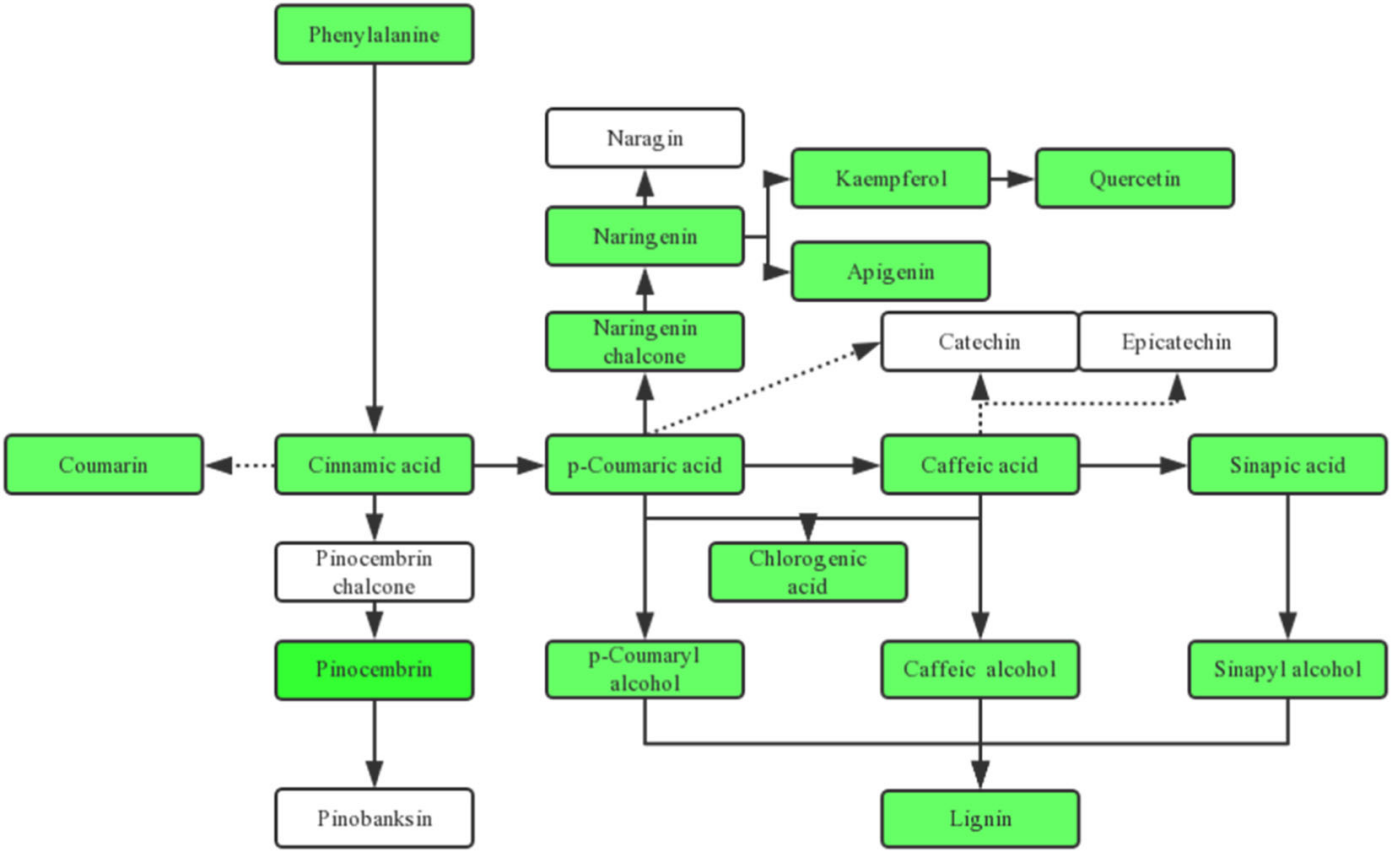
Differentially regulated metabolites related to the phenylpropanoid and flavonoid biosynthesis pathway under drought. The green color indicates metabolites downregulated in *MdMYB88/124* RNAi lines compared with those in GL-3 after long-term drought stress, and the white color represents metabolites that did not change in *MdMYB88/124* RNAi lines compared with GL-3 under drought stress

KEGG analysis was used to annotate the differentially regulated metabolites in the pathways of phenylpropanoid biosynthesis, flavonoid biosynthesis, and flavone and flavonol biosynthesis (Fig. 2 and Supplementary Figs. 2–4). All the differential metabolites in these three pathways were found to be downregulated in the *MdMYB88/124* RNAi plants under drought conditions compared to those in the GL-3 plants (Fig. 2). These metabolites included 15 involved in the phenylpropanoid biosynthesis pathway, 10 in the flavonoid biosynthesis pathway, and 7 in the flavone and flavonol biosynthesis pathway (Supplementary Figs. 2–4). We also discovered that the contents of 10 differential metabolites were downregulated in the *MdMYB88/124* RNAi plants under the control conditions compared to those in the GL-3 plants (Supplementary Figs. 2–4).

### MdMYB88 and MdMYB124 regulate the phenylalanine content in the roots

The KEGG analysis revealed a large number of differential metabolites related to the phenylpropanoid, flavonoid, flavone and flavonol biosynthesis pathways after drought stress (Fig. 2). Additionally, all these metabolites were downregulated in the roots of *MdMYB88/124* RNAi lines under drought (Supplementary Table 1); however, under control conditions, a smaller number of differential metabolites were discovered (Supplementary Table 1). These results indicate that MdMYB88 and MdMYB124 play a crucial role in the regulation of the phenylpropanoid, flavonoid, flavone, and flavonol biosynthesis pathways.

**Fig. 3:**
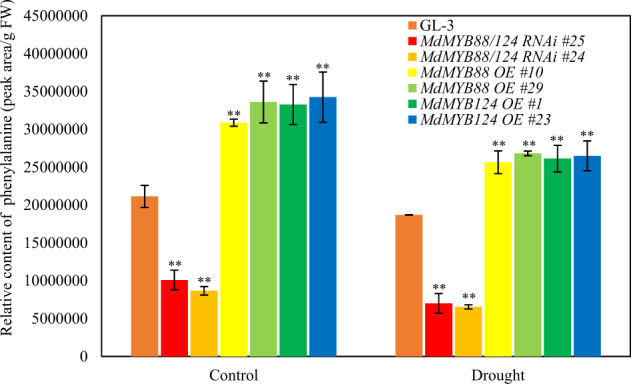
Content of phenylalanine in the roots of *MdMYB88* and *MdMYB124* transgenic plants after long-term drought. The data are the means ± SDs (*n* = 3). One-way ANOVA (Tukey test) was performed, and statistically significant differences are indicated by **(*P* < 0.01). OE overexpression

Furthermore, the content of phenylalanine in *MdMYB88/124* RNAi plants decreased under the control and drought conditions when compared with that in GL-3 plants (Supplementary Table 1). We therefore hypothesized that the downregulation of phenylpropanoids is the result of phenylalanine downregulation. Hence, we verified the content of phenylalanine in the roots of the *MdMYB88*/*124* RNAi lines and GL-3 under both drought and control conditions by the use of UPLC-MS (Fig. 3). Our results showed that the contents of phenylalanine in the roots of the *MdMYB88*/*124* RNAi lines were lower than those in GL-3 under both the control and long-term drought conditions. Alternatively, the phenylalanine content was higher in the roots of the overexpression lines than in GL-3 under both the control and drought conditions (Fig. 3). These findings were consistent with our metabolome results.

The differential content of phenylalanine is interesting. Biosynthesis of phenylalanine in plants occurs through the shikimic acid pathway. An essential reaction in phenylalanine biosynthesis is the conversion of chorismic acid into prephenic acid, catalyzed by CMs^9^. Three CMs have been discovered in *Arabidopsis*^42^, and six homologs in *Malus* x *domestica* were identified: MdCM1, MdCM1-like, MdCM2, MdCM2-like, MdCM3, and MdCM3-like (Supplementary Fig. 5). Sequence analysis of the *MdCM*s revealed a very high similarity between MdCM1 and MdCM1-like, between MdCM2 and MdCM2-like, and between MdCM3 and MdCM3-like. We also noted that MdCM1 is located on chromosome (chr.) 9, and MdCM1-like is located on chr. 16; MdCM2 is located on chr. 5, and MdCM2-like is located on chr. 8; MdCM3 is located on chr. 17, and MdCM3-like is located on chr. 13. The following pairs have homologous segments: chr. 9 and chr. 16, chr. 5 and chr. 8, and chr. 17 and chr. 13^43^. These results indicate that MdCM1 and MdCM1-like, MdCM2 and MdCM2-like, and MdCM3 and MdCM3-like are homologs from chromosome doubling.

**Fig. 4:**
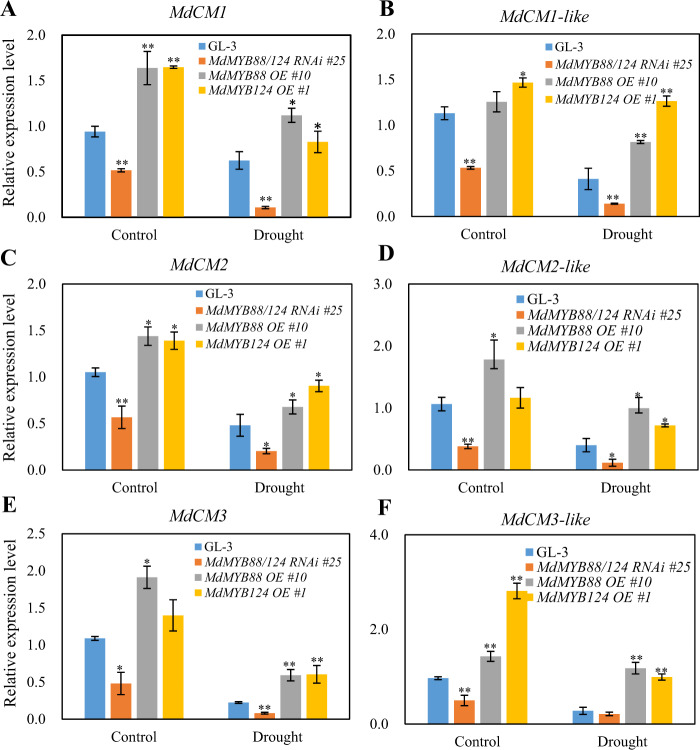
Relative expression levels of *MdCMs* in *MdMYB88* and *MdMYB124* transgenic plants under drought stress. Relative expression levels of *MdCM1* (**a**), *MdCM1-like* (**b**), *MdCM2* (**c**), *MdCM2-like* (**d**), *MdCM3* (**e**), and *MdCM3-like* (**f**) in *MdMYB88* and *MdMYB124* transgenic plants under control and drought conditions. The data are the means ± SDs (*n* = 3). OE overexpression

**Fig. 5:**
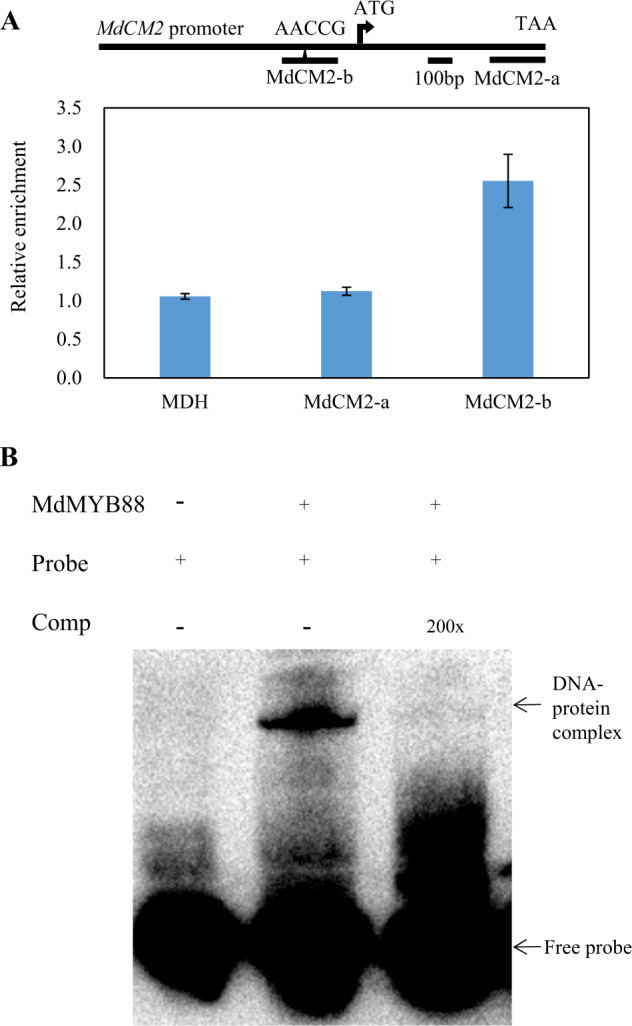
MdMYB88 and MdMYB124 directly target the *MdCM2* promoter. **a** ChiP-qPCR analysis of *MdCM2* using a native antibody against MdMYB88 and MdMYB124. Fragment *MdCM2*-a served as a negative control. Fragment *MdCM2*-b contains a *cis*-element of AACCG. The data are the means ± SDs (*n* = 3). **b** EMSA analysis of MdMYB88-His binding to the promoter region of *MdCM2*. The arrowheads indicate protein-DNA complex or free probe

The expression level of *MdCM*s in the roots of *MdMYB88* and *MdMYB124* transgenic lines treated with 20% PEG was analyzed by quantitative reverse transcription PCR (qRT-PCR) to determine whether *MdCM*s were regulated by MdMYB88 and MdMYB124 (Fig. 4). We found that all six homologs were downregulated in *MdMYB88/124* RNAi lines and unregulated in *MdMYB88* or *MdMYB124* overexpression lines under control and PEG treatment conditions. Promoter analysis revealed the binding site of both MdMYB88 and MdMYB124 (AACCG) in the *MdCM2* promoter (Supplementary Fig. 6). Using ChiP-qPCR and Electrophoretic Mobility Shift Assay (EMSA) approaches, we further showed that MdMYB88 can directly bind to the *MdCM2* promoter (Fig. 5).

### MdMYB88 and MdMYB124 regulate the content of different metabolites in the leaves

**Fig. 6:**
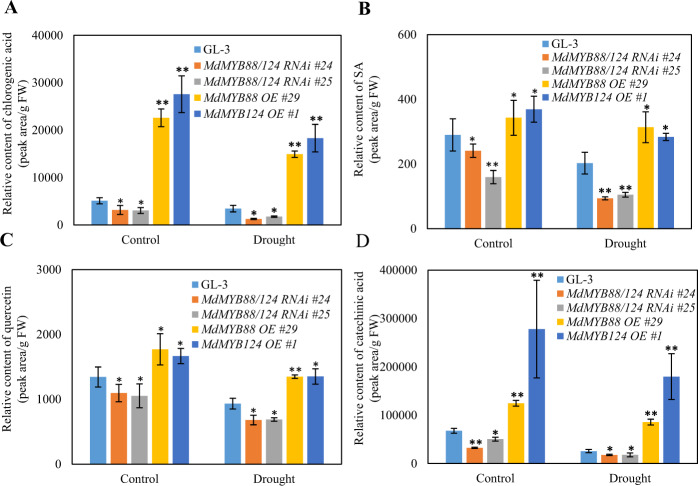
Content of phenylpropanoids in the leaves of *MdMYB88* and *MdMYB124* transgentic lines under drought stress. Content of chlorogenic acid (**a**), SA (**b**), quercetin (**c**), and catechinic acid (**d**) in the leaves under control and long-term drought stress conditions. The data are the means ± SDs (*n* = 3). One-way ANOVA (Tukey test) was performed, and statistically significant differences are indicated by *(*P* < 0.05) and **(*P* < 0.01)

*MdMYB88* and *MdMYB124* are also expressed in leaves^1^. We therefore examined the accumulation of different metabolites, including flavonoids and phenylpropanoids, in the leaves of the GL-3 and *MdMYB88*/*124* RNAi transgenic plants under both control and drought conditions via a UPLC-MS approach. Among the ten metabolites measured, the contents of chlorogenic acid, catechinic acid, quercetin, and SA (salicylic acid) decreased in the *MdMYB88*/*124* RNAi lines and increased in the *MdMYB88* or *MdMYB124* overexpression lines (Fig. 6). We found no significant differences in the metabolites, including gallic acid, phlorizin, rutin, and caffeic acid (Supplementary Fig. 7).

### Different contents of metabolites affect the occurrence of oxidative products and the resistance of pathogens

**Fig. 7:**
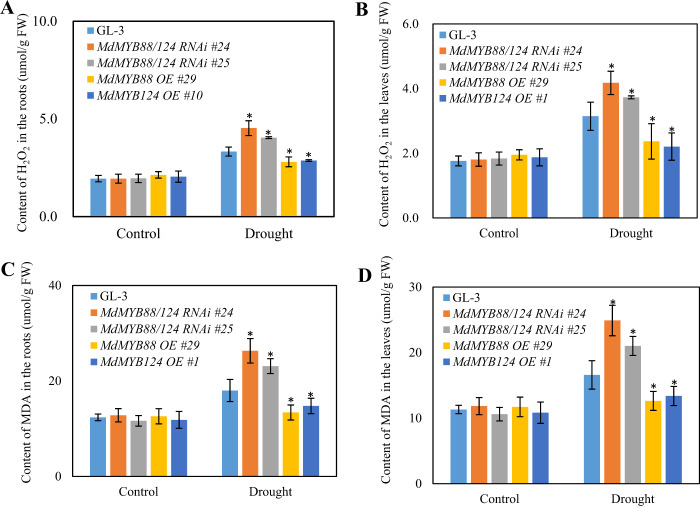
Content of H_2_O_2_ and MDA in *MdMYB88* and *MdMYB124* transgentic lines under drought stress. Content of H_2_O_2_ (**a** and **b**) and MDA (**c** and **d**) in the roots (**a** and **c**) and leaves (**b** and **d**) under control and drought stress conditions. The data are the means±SDs (*n* = 5). One-way ANOVA (Tukey test) was performed, and statistically significant differences are indicated by *(*P* < 0.05)

Flavonoid and phenylpropanoid metabolites have well-documented relationships with oxidative stress and pathogen resistance^44^. Drought stress is also well known to cause secondary stress, including oxidative stress^38^. We then analyzed the content of hydrogen peroxide (H_2_O_2_) and malondialdehyde (MDA) in both the roots and leaves of GL-3, *MdMYB88* and *MdMYB124* transgenic plants to examine the occurrence of drought-induced oxidative products. The results showed that there was no difference in the content of H_2_O_2_ or MDA between the GL-3 and transgenic lines under control conditions. There was, however, a significant increase in oxidative products in the *MdMYB88*/*124* RNAi lines and a decrease them in the *MdMYB88* or *MdMYB124* overexpression plants under drought stress (Fig. 7).

**Fig. 8:**
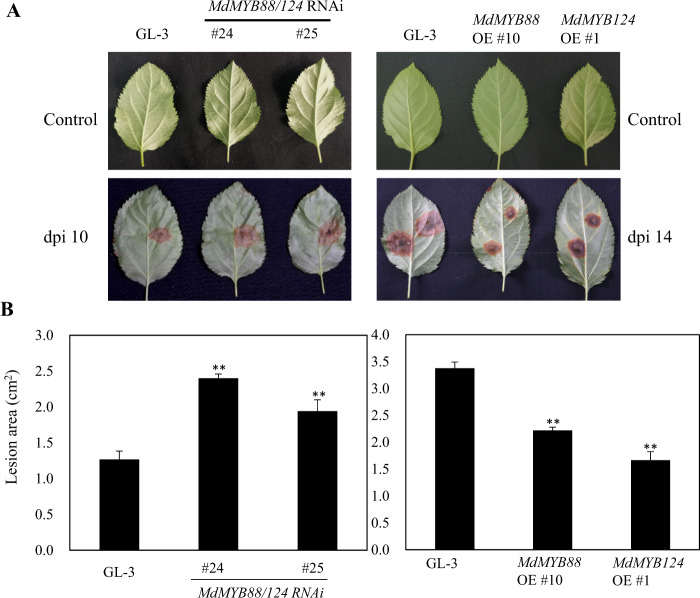
MdMYB88 and MdMYB124 positively regulate resistance to *Alternaria alternata* f. sp. *mali*. **a** Disease resistance of GL-3, *MdMYB88/124* RNAi, *MdMYB88*, and *MdMYB124* overexpression plants (OE). **b** Quantification of the data shown in **a**. The data are the means ± SDs (*n* = 8). One-way ANOVA (Tukey test) was performed, and statistically significant differences are indicated by **(*P* < 0.01)

**Fig. 9:**
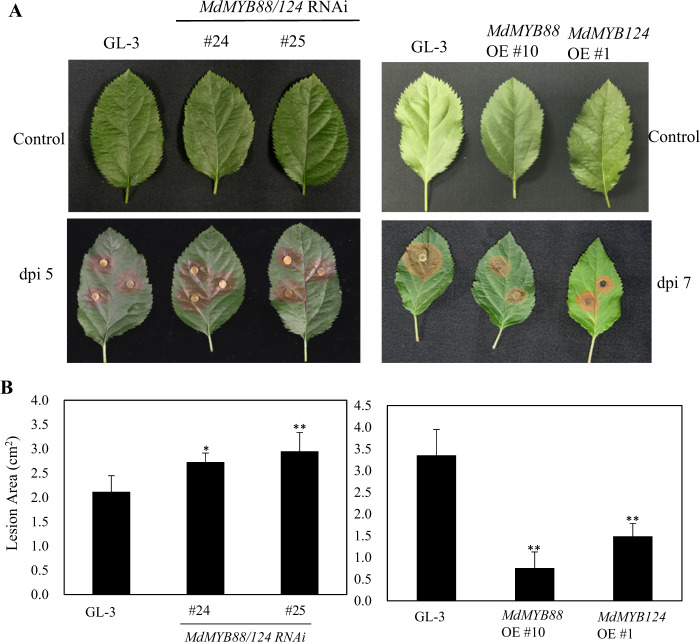
MdMYB88 and MdMYB124 positively regulate resistance to *Vasla mali*. **a** Disease resistance of GL-3, *MdMYB88/124* RNAi, *MdMYB88*, and *MdMYB124* overexpression plants (OE). **b** Quantification of the data shown in **a**. The data are the means ± SDs (*n* = 8). One-way ANOVA (Tukey test) was performed, and statistically significant differences are indicated by *(*P* < 0.05) or **(*P* < 0.01)

Flavonoid and phenylpropanoid metabolites are part of a plant’s defense system against pathogenic infection. The different contents of flavonoid and phenylpropanoid metabolites lead to differences in disease resistance^45^. We infected leaves using *A. alternata* and *Valsa mali* to evaluate the disease resistance of GL-3, *MdMYB88*, and *MdMYB124* transgenic plants *in vitro*. Our results demonstrate that the leaves of the *MdMYB88/124* RNAi lines were sensitive to *A. alternata*, while the leaves of the *MdMYB88* and *MdMYB124* overexpression lines were resistant to *A. alternata* compared with those of GL-3 (Fig. 8). We observed similar patterns in the leaves infected with *Valsa mali* (Fig. 9).

## Discussion

In previous studies, we presented evidence that MdMYB88 and MdMYB124 regulate both root vessel development and lignin deposition under drought stress in apple trees^2^. We also determined that MdMYB88 and MdMYB124 regulate the expression of genes related to lignin biosynthesis by associating with the *MdMYB46* promoter^2^. Lignin is a metabolic compound produced by the phenylpropanoid and flavonoid biosynthesis pathway^46^. In this study, we performed a metabolomic analysis of the roots of GL-3 and *MdMYB88/124* RNAi plants under control and drought conditions to understand the metabolic map of MdMYB88 and MdMYB124 and their regulatory roles in the disease resistance of apple trees.

The metabolome analysis revealed that more differentially accumulating metabolites were discovered in the roots of *MdMYB88*/*124* RNAi lines after drought treatment compared to those after the control treatment. A total of 46 different phenylpropanoid and flavonoid metabolites were discovered in the roots of *MdMYB88*/*124* RNAi plants under the control treatment, while 56 differential phenylpropanoid and flavonoid metabolites were discovered in the *MdMYB88/124* RNAi plants under the drought treatment (Supplementary Table 1). The majority of different phenylpropanoid and flavonoid metabolites were downregulated in *MdMYB88/124* RNAi plants compared to those in the GL-3 plants (Fig. 2). Furthermore, the content of lignin precursors (including p-coumaryl alcohol, coniferyl alcohol, and sinapyl alcohol) decreased in *MdMYB88/124* RNAi plants (Fig. 2). A decrease in the content of all three precursors can lead to a decreased lignin content in *MdMYB88/124* RNAi plants, which is consistent with our previous study^2^.

Many flavonoid metabolites, including chlorogenic acid^47^, quercetin^48^, kaempferol^49^, and pinocembrin^50^, are involved in a plant defense responses to fungal and bacterial infections. Derivatives of chlorogenic acid and chlorogenic acid can inhibit the growth of fungi in vivo and in vitro^51,52^. In response to fungal infection, chlorogenic acid in an apple plants can be hydrolyzed into 4-hydroxybenzoic acid, which has high antifungal activity, thus increasing the resistance of apple trees to fungi^53^. In immature peach trees and peach trees resistant to *Monilinia fructicola*, the chlorogenic acid content is exceptionally high^54^. Moreover, in vanilla plants resistant to *Fusarium oxysporum*, the sinapic acid and hydroxybenzoic acid content is higher than that in sensitive varieties^55^. Here, we discovered that these metabolites were positively regulated by MdMYB88 and MdMYB124, thus indicating that MdMYB88 and MdMYB124 may be involved in fungal resistance. We consistently found that the *MdMYB88*/*124* RNAi plants were more sensitive to *A. alternata* infection than were the other plants, while the *MdMYB88* and *MdMYB124* overexpression plants were more tolerant (Figs. 8 and 9).

The phenylpropanoid biosynthetic pathway starts with phenylalanine. CM2 is a critical enzyme involved in phenylalanine biosynthesis^42^. Herein, we determined that the expression of *MdCM2* and other *MdCM*s was positively regulated by MdMYB88 and MdMYB124 (Fig. 4). In addition, MdMYB88 and MdMYB124 could directly bind to the *MdCM2* promoter region (Fig. 5). These results revealed that MdMYB88 and MdMYB124 directly regulate *MdCM2*. However, we did not find any binding sites of MdMYB88 and MdMYB124 in the promoters of other *MdCM*s, although MdMYB88 and MdMYB124 positively regulate their expression level. These findings indicate that MdMYB88 and MdMYB124 may regulate other *MdCM*s through an unknown pathway.

In plants, different phenylpropanoid and flavonoid metabolite contents lead to a difference in the ability of plants to scavenge reactive oxygen species (ROS)^56^. In this study, the H_2_O_2_ and MDA contents were higher in *MdMYB88/124* RNAi plants under drought stress than in GL-3 plants; however, there was no significant difference between the GL-3 and the *MdMYB88* and *MdMYB124* transgenic lines under the control conditions (Fig. 6). These results indicate that the flavonoid content in the *MdMYB88* and *MdMYB124* transgenic lines influences the plants’ ROS scavenging abilities, which may further contribute to the drought tolerance of apple trees.

In summary, MdMYB88 and MdMYB124 can directly target and induce the expression of *MdCM2* and positively regulate other *MdCM*s. Upregulated *MdCM2* and other *MdCM*s enhance phenylalanine biosynthesis, thus increasing the content of free phenylalanine in both the roots and the leaves. The increased phenylalanine content affects the biosynthesis of downstream metabolites, including phenylpropanoid and flavonoid compounds, which results in increased resistance to oxidative stress and fungal infection.

## Methods

### Plant materials, growth conditions, and stress treatment

Tissue-cultured GL-3 [from *Malus* x *domestica* “Royal Gala” seedlings with high regeneration capabilties^57^], transgenic *MdMYB88/124* RNAi and MdMYB888 overexpression or MdMYB124 overexpression plants were rooted and transplanted into pots (30 cm × 18 cm) filled with equal parts of local loess sand and worm cast media. The pots were placed in a greenhouse under natural illumination, with a temperature of 20–35 °C and a humidity of 35–55%. *MdMYB88/124* RNAi plants were generated previously^1^. Owing to the high sequence similarity, both *MdMYB88* and *MdMYB124*^1^ were knocked down in the *MdMYB88/124* RNAi plants^1^.

Long-term drought was implemented according to the methods described by Geng et al*.*^2^. After drought stress, the roots were washed and harvested for UPLC-MS analysis.

For disease resistance analysis, GL-3 and *MdMYB88* or *MdMYB124* transgenic plants were propagated in MS culture media containing 0.2 mg/l 6-benzylaminopurine and 0.2 mg/l indole-3-acetic acid. After 1 month, the transgenic plants were transferred to rooting media containing 0.5 mg/l 3-indolebutyric acid and 0.5 mg/l indole-3-acetic acid. After 45 days, the plants were transplanted into soil and into a growth chamber with a 16/8-h light/dark photoperiod. After ~1 month, the apple plants were ready for the infection experiments.

### Disease resistance analysis

The plant pathogens *A. alternata* and *Valsa mali* were isolated previously^58^. The fungi were grown on PDA (potato dextrose agar) media at 25 °C for ~6 days. Fifteen to twenty leaves of GL-3 and MdMYB88 or *MdMYB124* transgenic plants were collected and washed with ddH_2_O twice, soaked in 6% sodium hypochlorite solution in the dark for approximately 5 min and then rewashed with ddH_2_O. The leaves were then infected using bacterial plaque obtained by a hole puncher to maintain consistency. After infection, the leaves were returned to the culture dishes and placed in an incubator (70% humidity; 25–28 °C) for two weeks. Afterward, images were taken, and the leaf spots were measured by ImageJ software. The experiments were repeated three times, and one-way ANOVA (Tukey test) was performed by SPSS 19.0.

### UPLC-MS analysis

UPLC-MS analysis was performed according to the methods of Chen et al.^59^, with modifications. Freeze-dried roots were crushed using a mixer mill (MM 400, Retsch, German) with silica sand for 2 min at 60 Hz. One hundred milligrams of powder was then weighed and extracted overnight at 4 °C with 1.0 ml of 70% aqueous methanol. Following centrifugation at 10,000 × *g* for 10 min, the extracts were absorbed (CNWBOND Carbon-GCB SPE Cartridge, 250 mg, 3 ml; Anpel, Shanghai, China) and filtered by a filter membrane with a pore size of 0.22 μm. The extracts were then analyzed using an Liquid chromatography electrospray ionization tandem mass spectrometry (LC-ESI-MS/MS) system. The analytical conditions were as follows: UPLC column, Waters ACQUITY UPLC HSS T3 C18 (1.8 µm, 2.1 mm × 100 mm); solvent system, water (0.04% acetic acid):acetonitrile (0.04% acetic acid); gradient program, 95:5 v/v at 0 min, 5:95 v/v at 11.0 min, 5:95 v/v at 12.0 min, 95:5 v/v at 12.1 min, and 95:5 V/Vv/v at 15.0 min; flow rate, 0.40 ml/min; temperature, 40 °C; and injection volume, 2 μl. The effluent was alternatively connected to an ESI-triple quadrupole-linear ion trap (QTRAP)-MS. LIT and triple quadrupole (QQQ) scans were acquired by a API 6500 QTRAP LC/MS/MS triple quadrupole-linear ion trap mass spectrometer (QTRAP) system. The ESI source operation parameters were the same as those in the study by Chen et al.^59^. PCA and Pearson correlation analysis were performed by SPSS 19.0. OPLS-DA, discovery and KEGG annotations were performed by the R package.

### Measurements of H_2_O_2_ and MDA content

The contents of H_2_O_2_ and MDA were measured by a hydrogen peroxide assay kit (#BC3590, Solarbio, Beijing, China) and a malondialdehyde assay kit (#BC0020, Solarbio, Beijing, China), respectively. Five repeats were measured for each line.

### RNA extraction and RT-qPCR analysis

RNA extraction was carried out as described by Xie et al.^1^. RT-qPCR analysis was performed according to the methods of Guan et al.^60^. The primers used are listed in Supplementary Table 2.

### EMSAs and ChIP-qPCR

EMSAs and ChIP-qPCR were performed as described by Xie et al.^1^. The probes used for the EMSAs are listed in Supplementary Table 2.

## Supplementary information

supplemental table 2

supplemental figures

supplemental table 1
