# Supplementary material for: Regulation of phenylpropanoid biosynthesis by MdMYB88 and MdMYB124 contributes to pathogen and drought resistance in apple
Source: Hortic Res. 2020 Jul 1;7:102. doi: 10.1038/s41438-020-0324-2 (PMC7327078; doi:10.1038/s41438-020-0324-2)
Supplement: Supplementary file 1 — supplemental table 2 [file 41438_2020_324_MOESM1_ESM.pdf]

**Supplemental Table 2.** Primers used in experiment.		
Primer Name	Primer Sequence (5' to 3')	Purpose
MdCM1F	AGGCTCTGTCCAAGCGAATC	qRT-PCR analysis
MdCM1R	TGATTGCCTCCTCAACCGTC	
MdCM1likeF	TTTCCCCGTTGATGGGTTCC	qRT-PCR analysis
MdCM1likeR	AATGGGATGCAGAACCTGGG	
MdCM2F	GGGTTCCCCTCAAGACTACG	qRT-PCR analysis
MdCM2R	CTTGCGTTGTGTTTGTCGGT	
MdCM2like F	CCCGGAAAGTTTACCGCCTT	qRT-PCR analysis
MdCM2like R	GCAGTTGACGCATAGCTTCC	
MdCM3F	GCAAGCTTCAATTCCCAATGCT	qRT-PCR analysis
MdCM3R	GAAGAAACTTGGAGAGGACGG	
MdCM3likeF	GTGGCAGAGGCAAAATTCCG	qRT-PCR analysis
MdCM3likeR	CTGCCCGTATCGTTTCGTCT	
MdCM2-probe-bioF	CCCCACTAACCGAGAAATCCCCCACTAACCGAGAAATCC CCCACTAACCGAGAAATCCCCCACTAACCGAGAAATC	Biotin-labeled (5'-end) or unlabled probe of MdCM2 promoter cis-element for EMSAs
MdCM2-probe-bioR	GATTTCTCGGTTAGTGGGGGATTTCTCGGTTAGTGGGG GATTTCTCGGTTAGTGGGGGATTTCTCGGTTAGTGGGG	
MdCM2-probe-unbioF	CCCCACTAACCGAGAAATCCCCCACTAACCGAGAAA TCCCCCACTAACCGAGAAATCCCCCACTAACCGAGAAATC	Unlabled probe of MdCM2 promoter cis-element for EMSAs
MdCM2-probe-unbioR	GATTTCTCGGTTAGTGGGGGATTTCTCGGTTAGTGGGGG ATTTCTCGGTTAGTGGGGGATTTCTCGGTTAGTGGGG	
MdCM2-chipqPCR-aF	AGGGCAAGAAGTAAGCCT	Chip-qPCR of MdCM2 promoter fragement a
MdCM2-chipqPCR-aR	CTAGTCTAGGCGGCGAAGG	
MdCM2-chipqPCR-bF	TAAAATTCGGTGAACCCGCGT	Chip-qPCR of MdCM2 promoter fragement b
MdCM2-chipqPCR-bR	GTGGTGGTGGAGGGTT	
