# Supplementary material for: Regulation of phenylpropanoid biosynthesis by MdMYB88 and MdMYB124 contributes to pathogen and drought resistance in apple
Source: Hortic Res. 2020 Jul 1;7:102. doi: 10.1038/s41438-020-0324-2 (PMC7327078; doi:10.1038/s41438-020-0324-2)
Supplement: Supplementary file 2 — supplemental figures [file 41438_2020_324_MOESM2_ESM.pptx]

## Slide 1
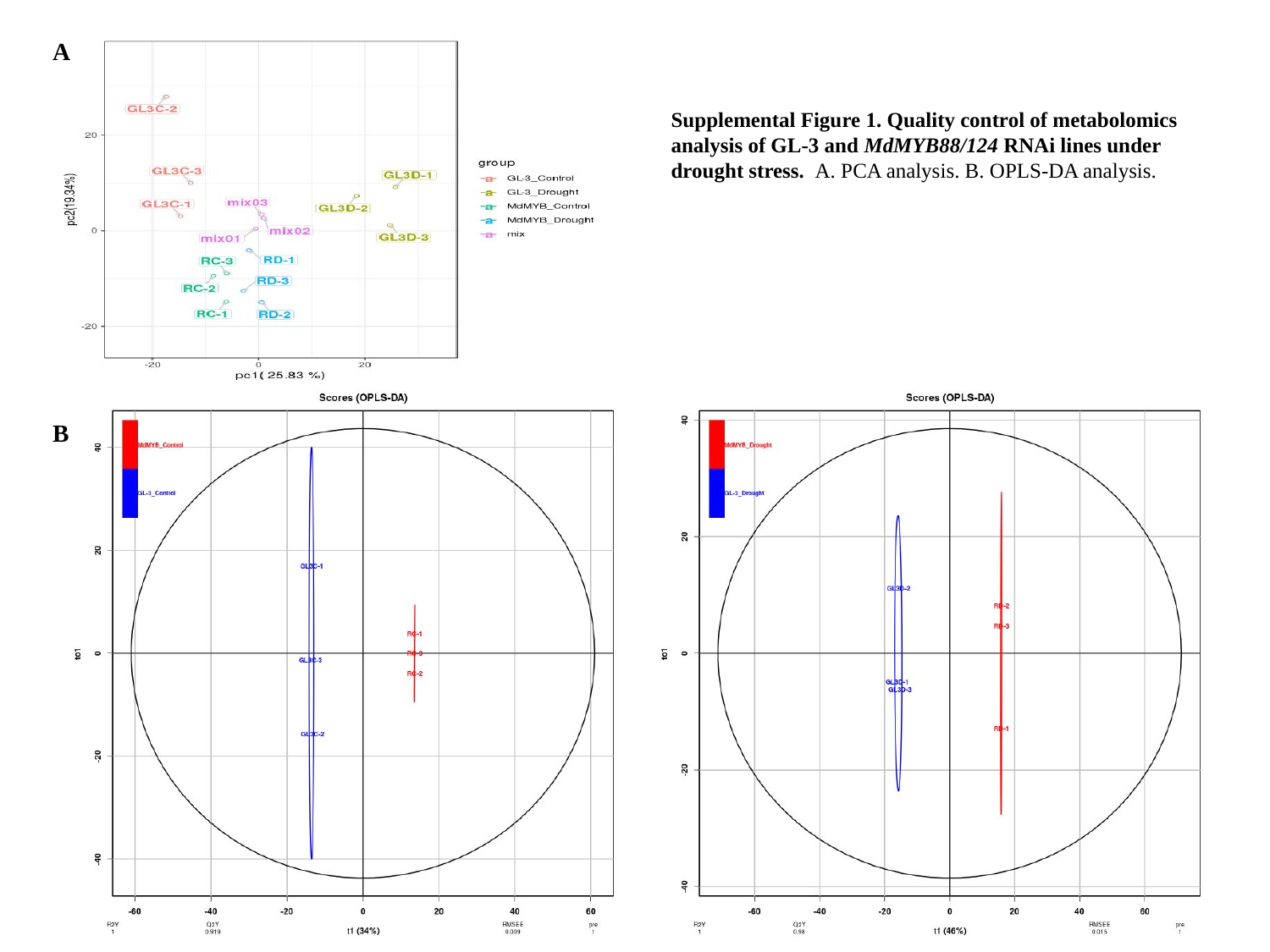

A
Supplemental Figure 1. Quality control of metabolomics analysis of GL-3 and MdMYB88/124 RNAi lines under drought stress.  A. PCA analysis. B. OPLS-DA analysis.
B

## Slide 2
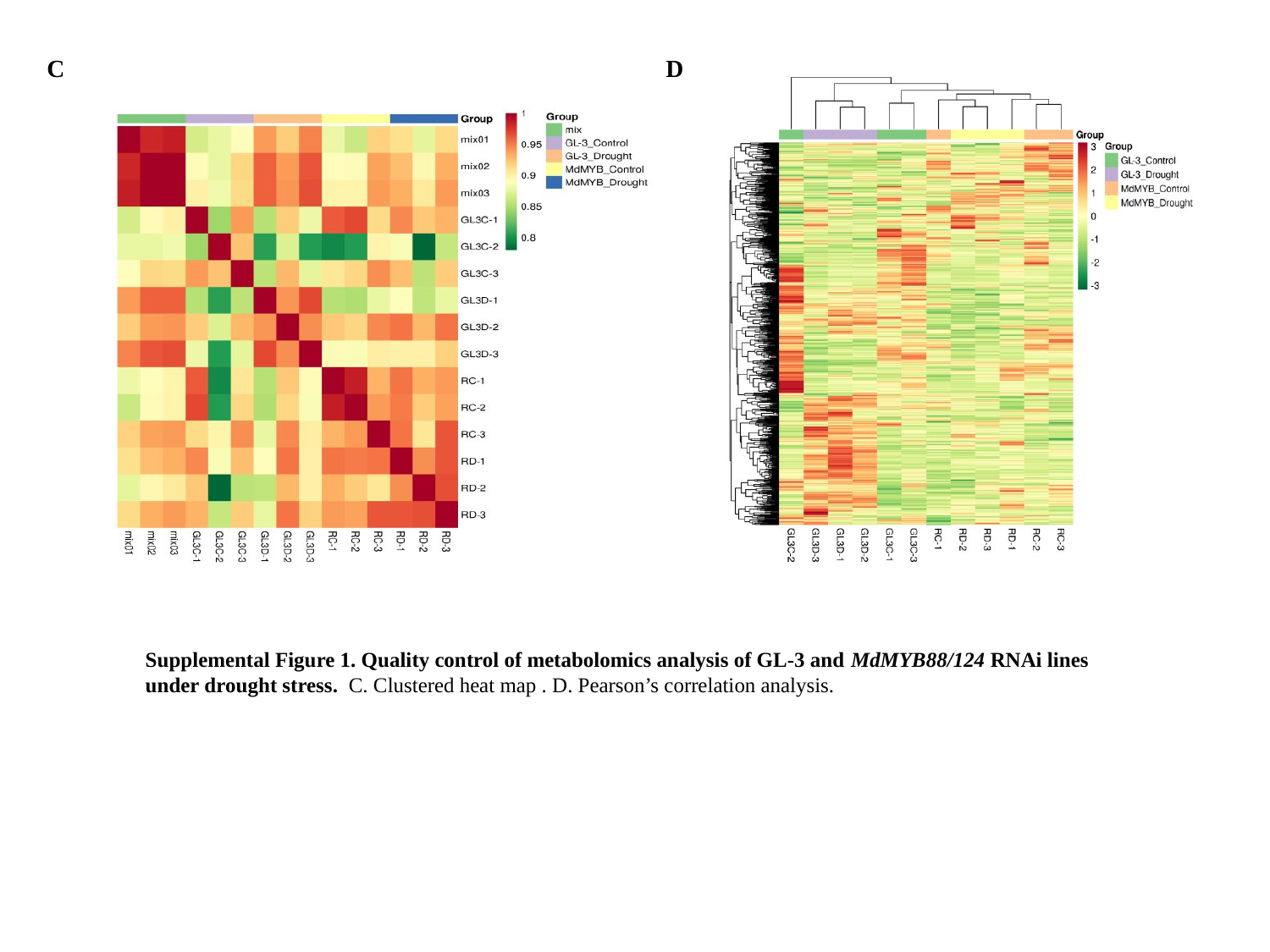

C
D
Supplemental Figure 1. Quality control of metabolomics analysis of GL-3 and MdMYB88/124 RNAi lines under drought stress.  C. Clustered heat map . D. Pearson’s correlation analysis.

## Slide 3
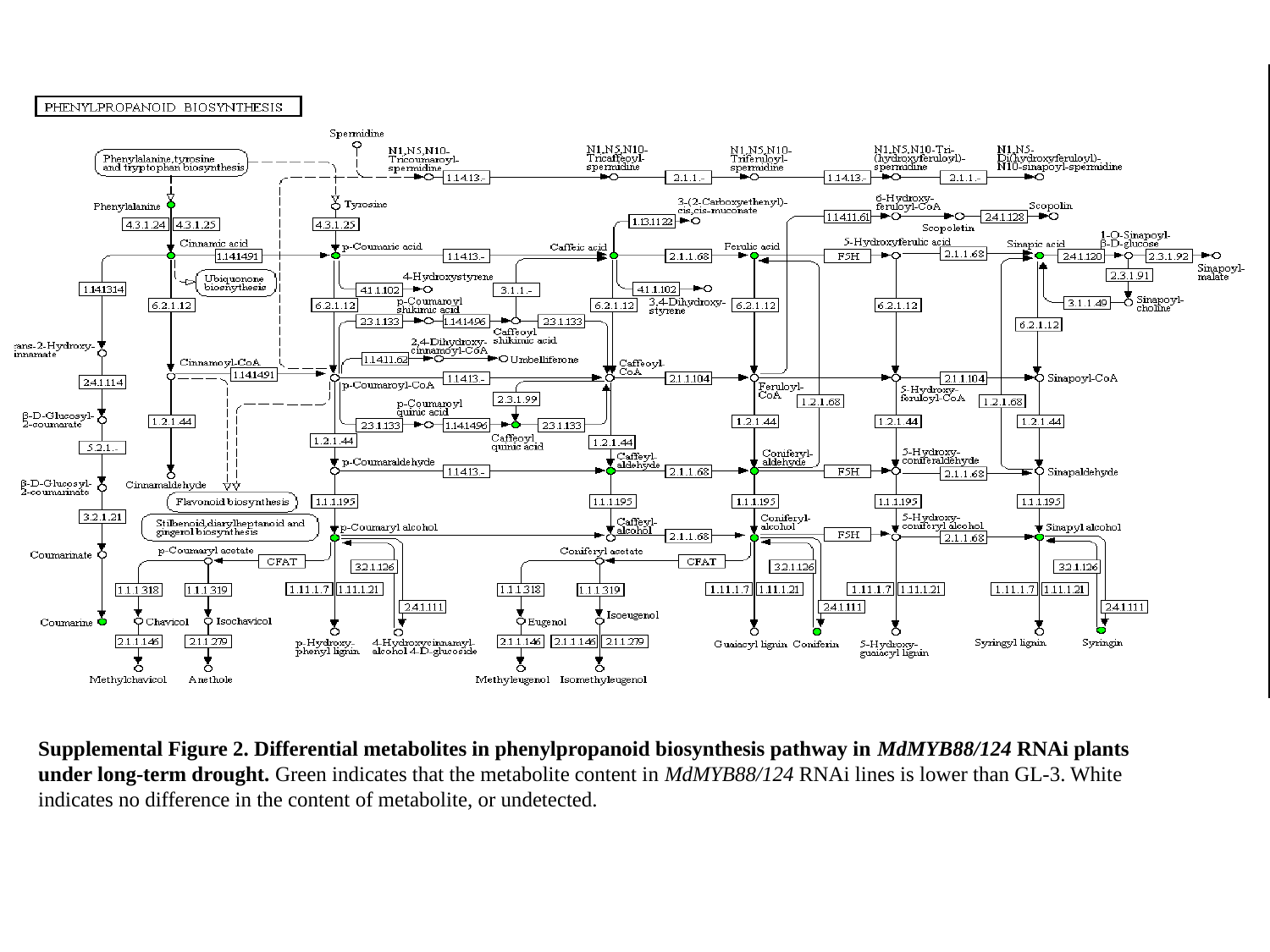

Supplemental Figure 2. Differential metabolites in phenylpropanoid biosynthesis pathway in MdMYB88/124 RNAi plants under long-term drought. Green indicates that the metabolite content in MdMYB88/124 RNAi lines is lower than GL-3. White indicates no difference in the content of metabolite, or undetected.

## Slide 4
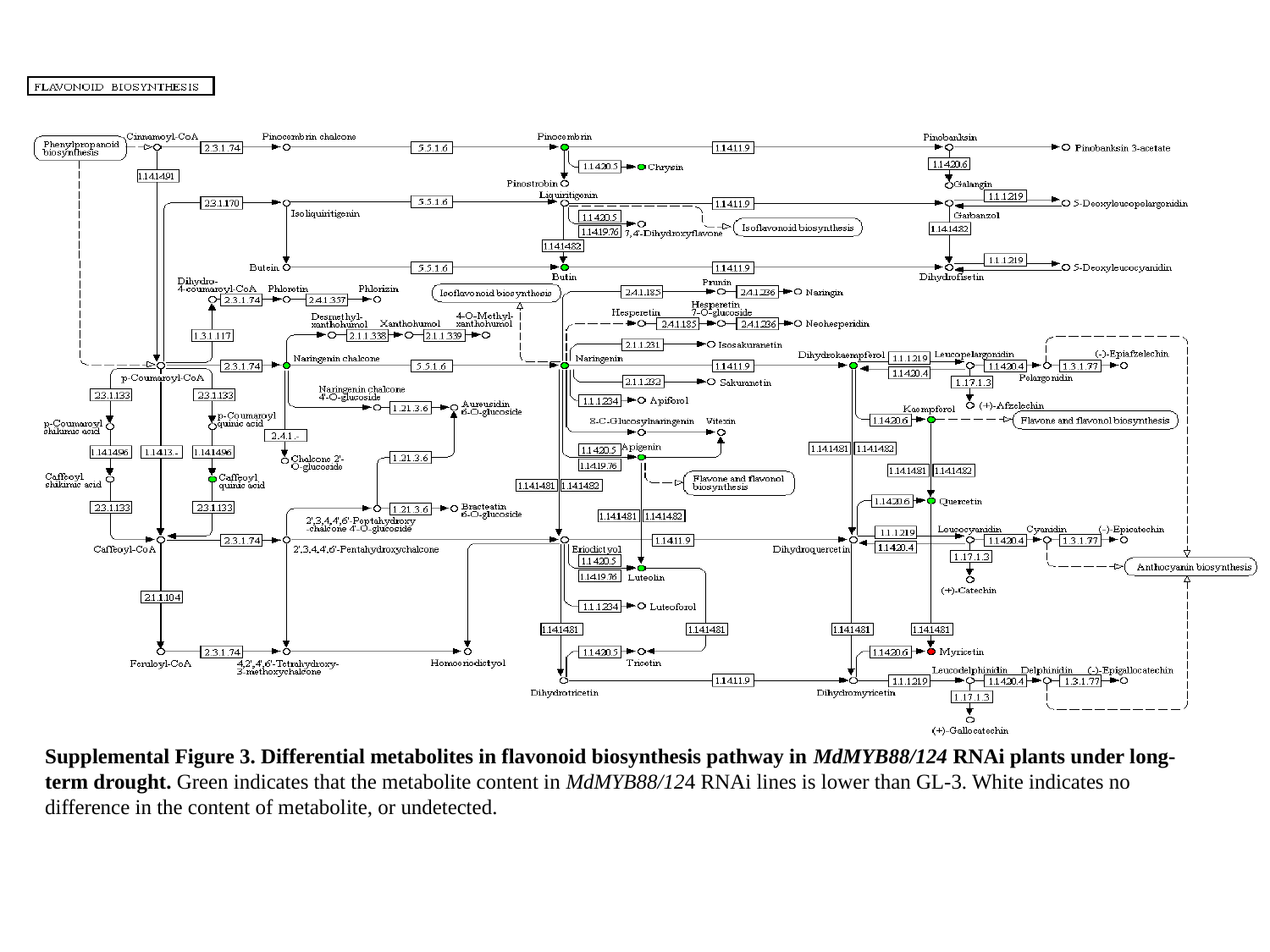

Supplemental Figure 3. Differential metabolites in flavonoid biosynthesis pathway in MdMYB88/124 RNAi plants under long-term drought. Green indicates that the metabolite content in MdMYB88/124 RNAi lines is lower than GL-3. White indicates no difference in the content of metabolite, or undetected.

## Slide 5
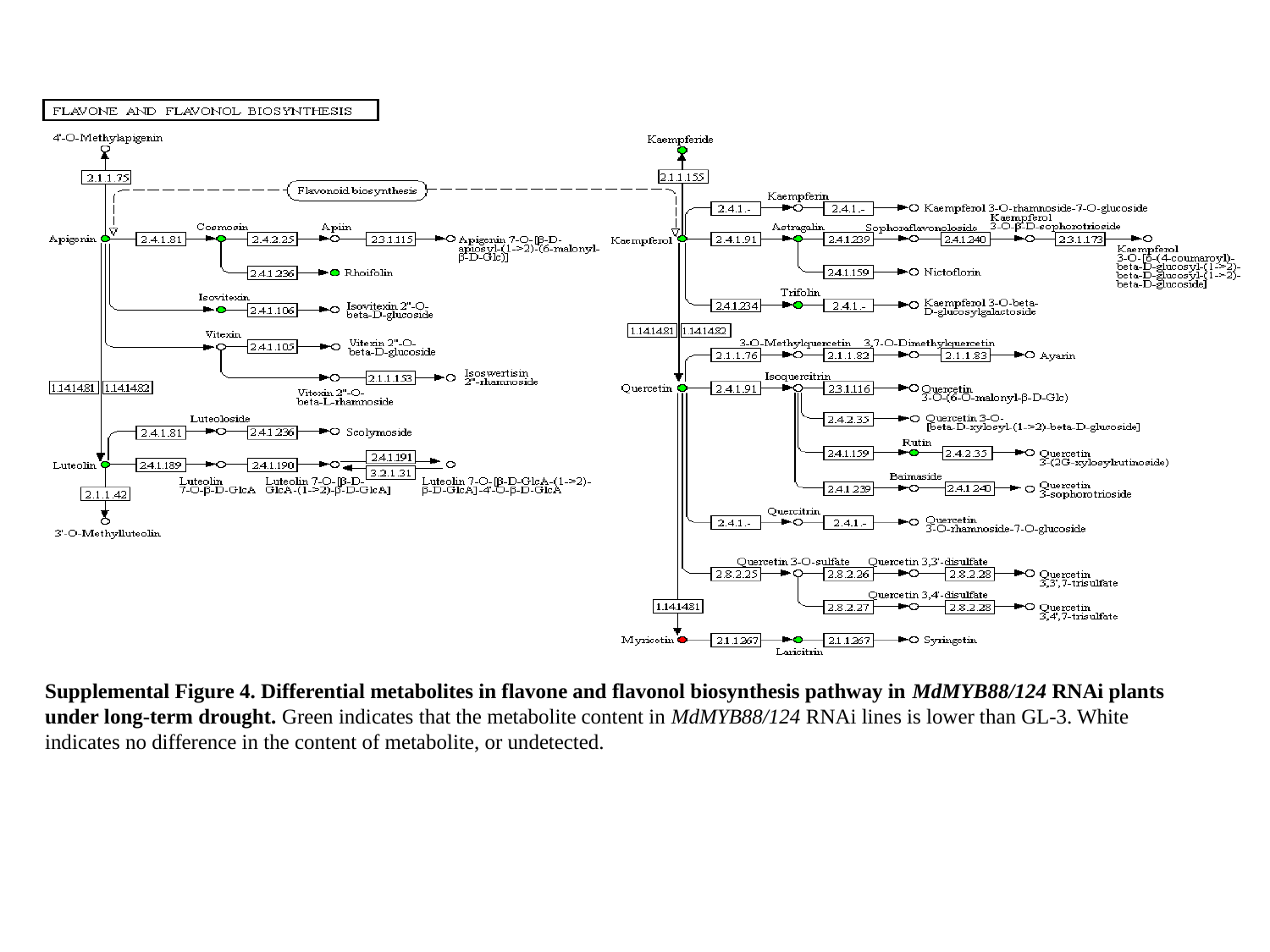

Supplemental Figure 4. Differential metabolites in flavone and flavonol biosynthesis pathway in MdMYB88/124 RNAi plants under long-term drought. Green indicates that the metabolite content in MdMYB88/124 RNAi lines is lower than GL-3. White indicates no difference in the content of metabolite, or undetected.

## Slide 6
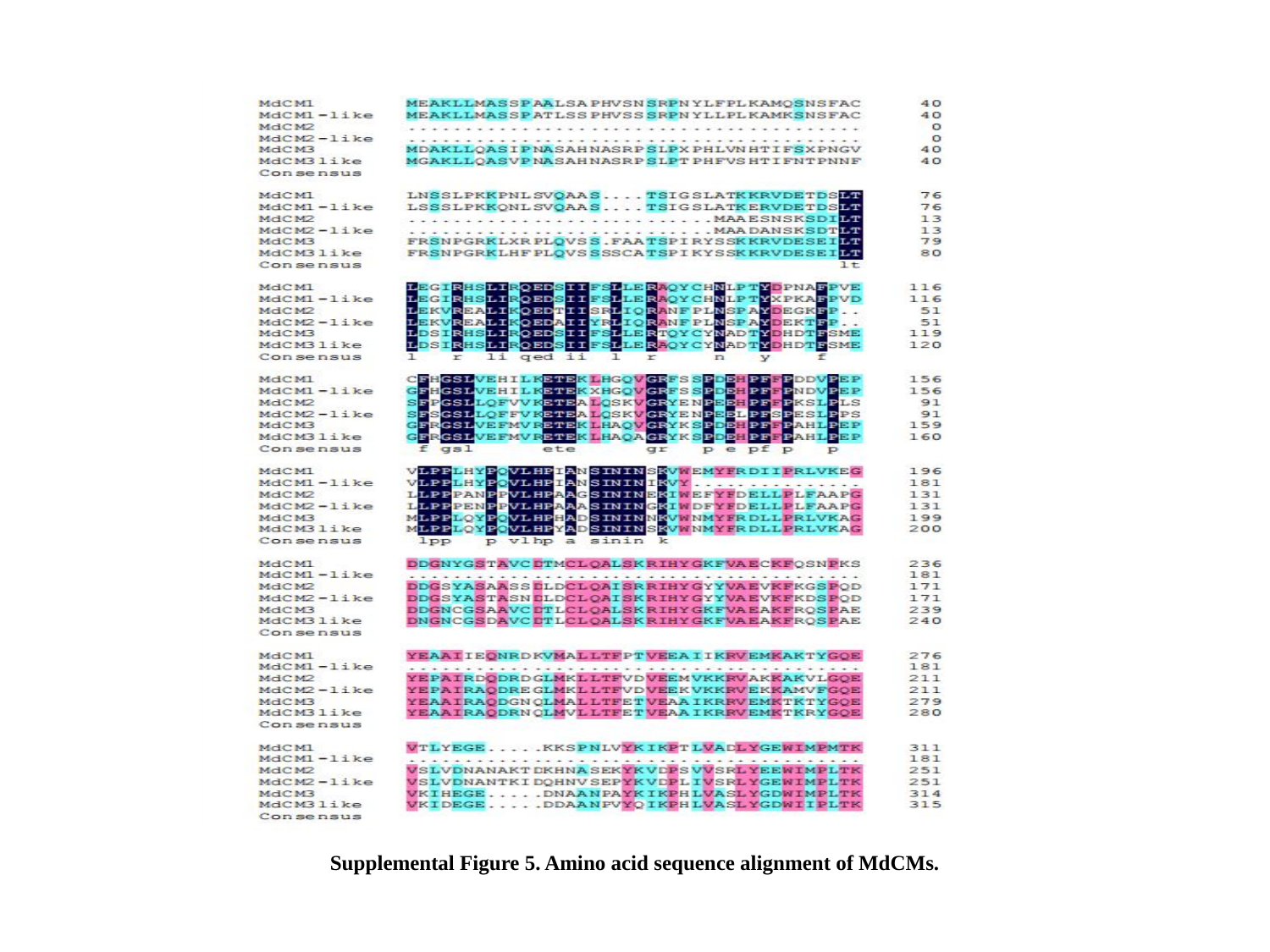

Supplemental Figure 5. Amino acid sequence alignment of MdCMs.

## Slide 7
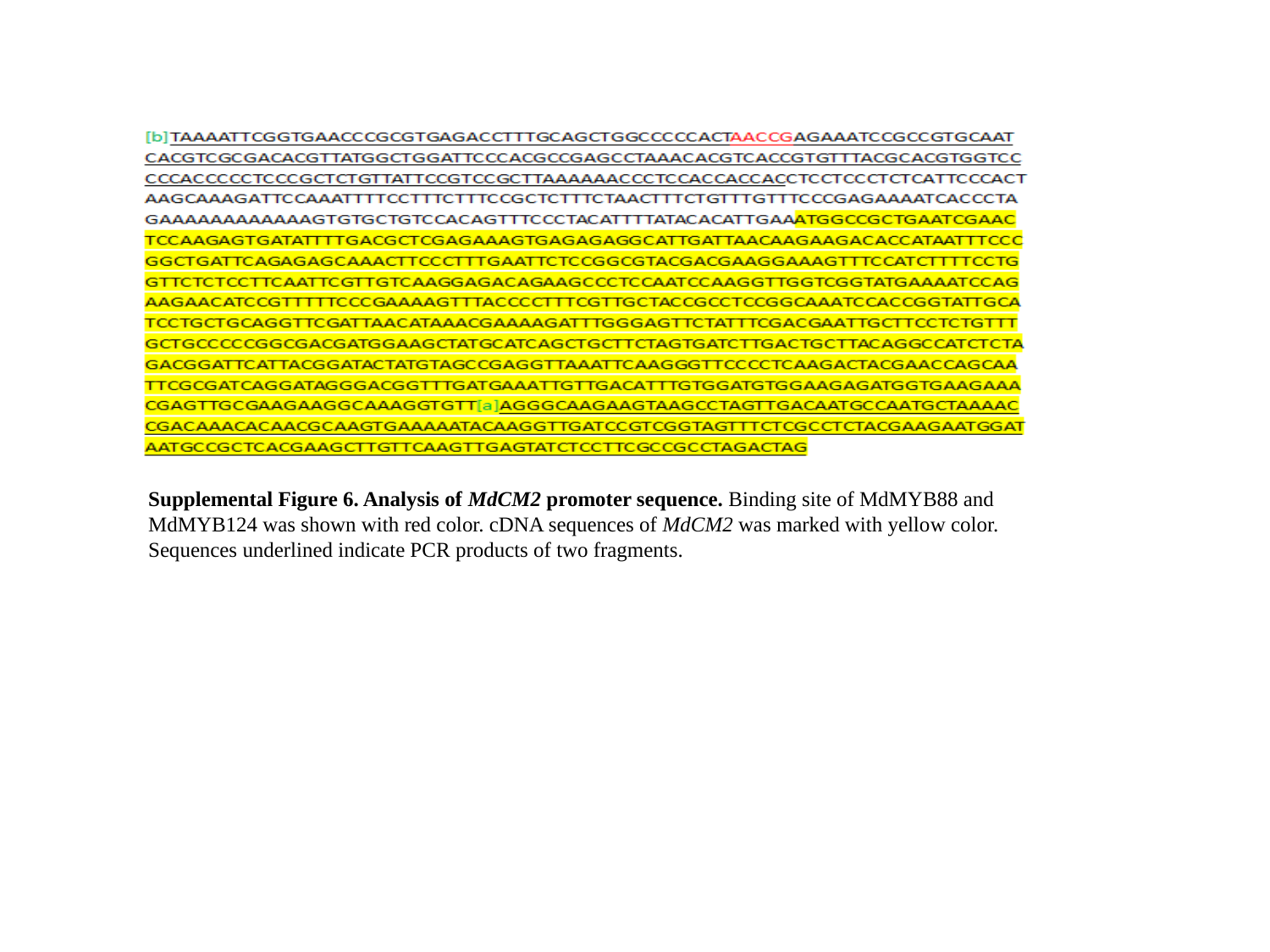

Supplemental Figure 6. Analysis of MdCM2 promoter sequence. Binding site of MdMYB88 and MdMYB124 was shown with red color. cDNA sequences of MdCM2 was marked with yellow color. Sequences underlined indicate PCR products of two fragments.

## Slide 8
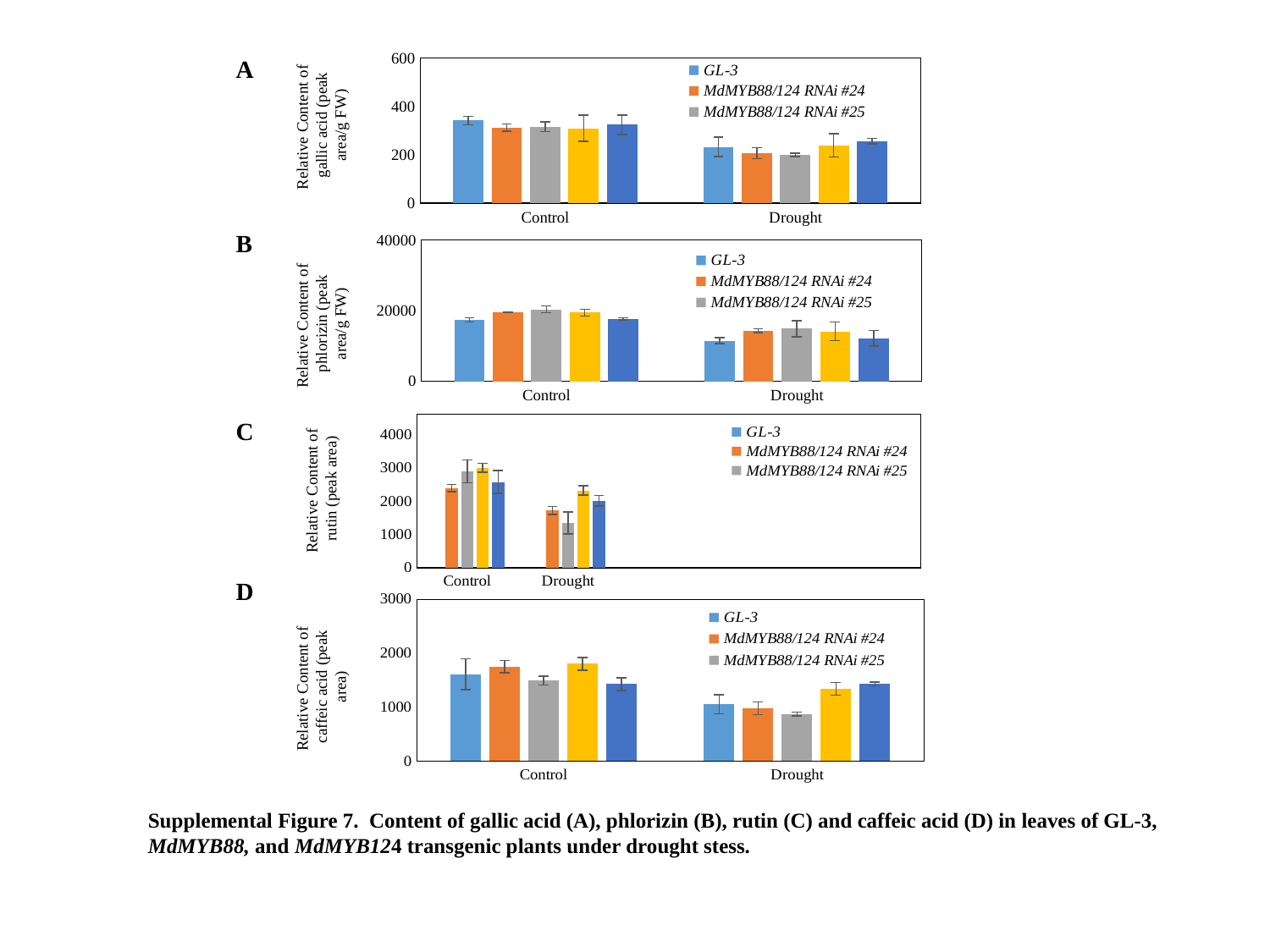

### Chart
| Category | GL-3 | MdMYB88/124 RNAi #24 | MdMYB88/124 RNAi #25 | MdMYB88 OE #29 | MdMYB124 OE #10 |
|---|---|---|---|---|---|
| Control | 342.062593333333 | 313.13342 | 316.91129 | 309.617516666667 | 324.590996666667 |
| Drought | 232.953826666667 | 206.933856666667 | 200.032156666667 | 239.0097 | 256.88065 |A
Relative Content of gallic acid (peak area/g FW)
B
### Chart
| Category | GL-3 | MdMYB88/124 RNAi #24 | MdMYB88/124 RNAi #25 | MdMYB88 OE #29 | MdMYB124 OE #10 |
|---|---|---|---|---|---|
| Control | 17381.9368733333 | 19577.4625933333 | 20371.8716966667 | 19459.0586133333 | 17688.6278633333 |
| Drought | 11533.83338 | 14384.5707166667 | 14943.2178433333 | 14167.28822 | 12135.35758 |Relative Content of phlorizin (peak area/g FW)
### Chart
| Category | GL-3 | MdMYB88/124 RNAi #24 | MdMYB88/124 RNAi #25 | MdMYB88 OE #29 | MdMYB124 OE #10 |
|---|---|---|---|---|---|
| Control | 2221.78280333333 | 2388.37197 | 2890.52837333333 | 3000.82362 | 2570.04439333333 |
| Drought | 1756.73195333333 | 1717.91035666666 | 1340.87253333333 | 2317.0307 | 2011.03305 |C
Relative Content of rutin (peak area)
D
### Chart
| Category | GL-3 | MdMYB88/124 RNAi #24 | MdMYB88/124 RNAi #25 | MdMYB88 OE #29 | MdMYB124 OE #10 |
|---|---|---|---|---|---|
| Control | 1608.0607 | 1752.01031 | 1491.60641 | 1801.18464666667 | 1424.62413 |
| Drought | 1050.35293333333 | 976.46021 | 874.38853 | 1337.11352666667 | 1428.29153333333 |Relative Content of caffeic acid (peak area)
Supplemental Figure 7. Content of gallic acid (A), phlorizin (B), rutin (C) and caffeic acid (D) in leaves of GL-3, MdMYB88, and MdMYB124 transgenic plants under drought stess.
